# Effect of thoracic paraspinal block-propofol intravenous general anesthesia on VEGF and TGF-β in patients receiving radical resection of lung cancer

**DOI:** 10.1097/MD.0000000000018088

**Published:** 2019-11-22

**Authors:** Yang Sen, Hu Xiyang, Han Yu

**Affiliations:** a First Department of Anesthesia; b Department of Radiology; c Nuclear Medicine Department, Cangzhou Central Hospital, Hebei Province, China.

**Keywords:** lung cancer, propofol, TGF-β, thoracic paraspinal nerve block, VEGF

## Abstract

The objective of this study is to compare the effects of paravertebral nerve block-propofol intravenous general anesthesia (PPA) and sevoflurane inhalation general anesthesia (SGA) on the expression of serum vascular endothelial growth factor (VEGF) and transforming growth factor beta (TGF-β) in patients undergoing radical resection of lung cancer.

Patients undergoing radical resection of lung cancer were divided into PPA group and SGA group. In PPA group, thoracic paraspinal nerve block was performed with 0.5% ropivacaine (2 mg/kg) before general anesthesia. Anesthesia was maintained with 2.5–3.5 μg/mL TCI of propofol. In SGA group, anesthesia was maintained with 1.0–1.5 MAC sevoflurane. The dosage of opioids during and 24 h after operation, the pain score at 2, 8, 24, 48, and 72 h after operation, and the concentrations of serum VEGF and TGF-β before and 24 h after operation were observed in the two groups.

The intraoperative dosage of remifentanil in PPA group was significantly less than that in SGA group (*P* < 0.05). The dosage of sufentanil in SGA group was significantly less than that in SGA group at 24 h after operation (*P* < 0.05). The VAS score at 2, 8, and 24 h after operation was significantly lower than that in SGA group (*P* < 0.05). The serum VEGF and TGF-β concentration in PPA group was significantly lower than that in SGA group (*P* < 0.05).

Thoracic paravertebral nerve block-propofol intravenous general anesthesia can reduce the dosage of opioids, improve the effect of postoperative analgesia, and reduce the serum concentration of tumor angiogenesis-related factors in patients undergoing radical resection of lung cancer.

## 1 Introduction

Lung cancer is the most common malignant tumor in the world, and it is the main cause of cancer death in developed countries.^[1]^ Surgery is an important method for the treatment of lung cancer, but postoperative recurrence and metastasis are the factors affecting the survival rate.^[2]^ Perioperative anesthesia management is closely related to postoperative rehabilitation, and may even be associated with long-term prognosis of the tumor.^[3]^ Previous studies have shown that different anesthetics and anesthesia methods affect perioperative immune function, tumor cell proliferation, invasion, and metastasis, and thus affect the long-term prognosis of patients.^[4]^ There are few studies on the effect of anesthesia on postoperative recurrence and metastasis of non-small cell lung cancer (NSCLC). Serum vascular endothelial growth factor (VEGF) and transforming growth factor beta (TGF-β) promote angiogenesis in lung cancer and play an important role in the pathogenesis, progression and metastasis of NSCLC.^[5]^ This study was performed in patients undergoing thoracoscopic-assisted radical resection of NSCLC. The paravertebral nerve block-propofol intravenous general anesthesia (PPA) or sevoflurane inhalation general anesthesia (SGA) were performed during operation. We observed the effects of different anesthetic methods on perioperative opioid dosage, postoperative visual simulation score (VAS), and serum VEGF and TGF-β concentrations. It is expected to provide a clinical basis for patients with NSCLC to choose the appropriate mode of anesthesia.

## 2 Materials and methods

### 2.1 Subjects

This was a single-center prospective study evaluating the effects of different anesthetic methods on the expression of VEGF and TGF-β in patients undergoing radical resection of NSCLC. This study was approved by the Institutional Review Board of Cangzhou Central Hospital and was conducted in accordance with the contents of the Declaration of Helsinki. All patients signed informed consent. From January 2016 to December 2018, 90 patients receiving thoracoscopic-assisted radical resection of NSCLC were divided into two groups by random number table: PPA group and SGA group.

Criteria for selecting the subjects were as follows: age 40–70 years old, patients with early stage NSCLC were scheduled to undergo thoracoscopic-assisted radical resection, KPS > 70, and normal cardiac, hepatic, and renal function.

The exclusion criteria were as follows: patients receiving hormones, immunosuppressive drugs, radiotherapy, or chemotherapy before operation, patients with spinal deformities, back infections, etc. are not suitable for parathoracic block, postoperative pathological results were benign lesions, patients underwent thoracotomy for various reasons, patients bled more than 800 mL or needed intraoperative blood transfusion, and patients had severe toxic reactions of local anesthetics.

### 2.2 Thoracic paravertebral nerve block

The patients in PPA group were given 1–2 mg midazolam and 5 μg sufentanil for sedation and analgesia. After routine disinfection and towel laying, 4/5 and 6/7 paravertebral nerve block were performed under ultrasound guidance by the same senior physician. 0.5% ropivacaine was injected into the thoracic paravertebral nerve according to the dosage of 2 mg/kg after withdrawal of blood and gas. The block plane was measured after 15 min, followed by anesthesia induction.

### 2.3 Induction of anesthesia

0.04–0.06 mg/kg midazolam, 0.3–0.5 ug/kg sufentanil, 0.2–0.3 mg/kg etomidate, and 0.2 mg/kg cis-atracurium were injected intravenously, and mechanical ventilation was performed after insertion of double-lumen tracheal tube. The tidal volume was 8 mL/kg and the respiratory rate was 12–14 times/min during bilateral lung ventilation. During single-lung ventilation, tidal volume was 6 mL/kg, frequency was 15-17 times/min, the respiratory ratio was 1:1.5, oxygen flow was 2 L/min, and end-breathing carbon dioxide was maintained at about 35 mmHg.

### 2.4 Anesthesia maintenance

In the PPA group, anesthesia was maintained with 2.5–3.5 μg/mL TCI of propofol. In the SGA group, anesthesia was maintained with 1.0–1.5 MAC sevoflurane. Remifentanil and cis-atracurium were used to maintain the heart rate and blood pressure at about 20% of the preoperative baseline value, and BIS was between 45 and 60. During the operation, the dosage of remifentanil, sevoflurane and propofol was adjusted according to BIS value, blood pressure, and heart rate.

### 2.5 Postoperative analgesia

Multimodal analgesia was applied after operation. Flurbiprofen was given intravenously 100 mg twice a day. 150 μg sufentanil and 4 mg ondansetron were dissolved in 150 mL saline solution and pumped intravenously at a rate of 2 mL/h by micro-pump.

### 2.6 Sample collection

4 mL peripheral venous blood was collected before operation and 24 h after operation. The samples were centrifuged at 4000 g for 10 min. The levels of serum VEGF and TGF-β were quantitatively measured by commercially available enzyme-linked immunosorbent assay (ELISA) kits according to the manufacturer's instructions.

### 2.7 Observation indicators

The consumption of remifentanil during operation and sufentanil after operation, VAS score at 2, 8, 24, 48, and 72 h after operation and adverse reactions, as well as the serum levels of VEGF and TGF-β before and after operation were observed.

### 2.8 Statistical analysis

The statistical analysis was carried out by SPSS22.0 software. The measurement data were expressed as mean ± standard deviation. The independent sample t test was used to compare the two samples. The result between pre-and post-operation was compared with matched pair t test. The qualitative data were expressed by frequency and analyzed by chi-square test. Statistical significance was set at a *P*-value of less than 0.05.

## 3 Results

### 3.1 Demographics

In PPA group, two patients were diagnosed as benign lesions by postoperative pathology and two patients were given thoracotomy due to thoracic adhesions. In SGA group, two patients were diagnosed as benign lesions, one patient received thoracotomy because of thoracic adhesions, and one patient was excluded due to intraoperative blood transfusion. Finally, 41 patients were enrolled in each group. All anesthesia and surgical procedures are performed by the same group of anesthesiologists and surgeons. There was no significant difference in age, sex, BMI, operation time, pathological type and tumor stage between the two groups (*P* > 0.05), as shown in Table 1.

**Table 1:**
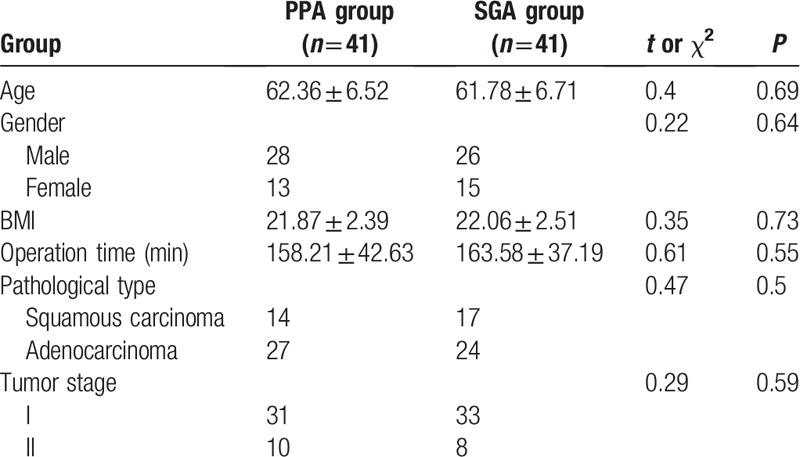
Patients demographics and clinical data in two groups.

### 3.2 Comparison of the consumption of analgesics between two groups

Intraoperative remifentanil consumption in PPA group was significantly lower than that in SGA group (*P* < 0.05). Sufentanil consumption in PPA group was significantly lower than that in SGA group within 24 hours after operation (*P* < 0.05), as shown in Table 2.

**Table 2:**
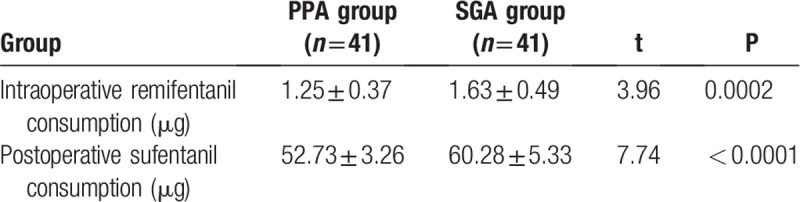
Comparison of the consumption of analgesics between two groups.

### 3.3 Comparison of postoperative VAS scores between two groups

There was significant difference in VAS scores at different time point in two groups (*P* < 0.05). VAS score of PPA group was significantly lower than that of SGA group at 2, 8, and 24 h after operation (*P* < 0.05), but there was no significant difference in VAS scores between two groups at 48 and 72 h after operation, as shown in Table 3.

**Table 3:**
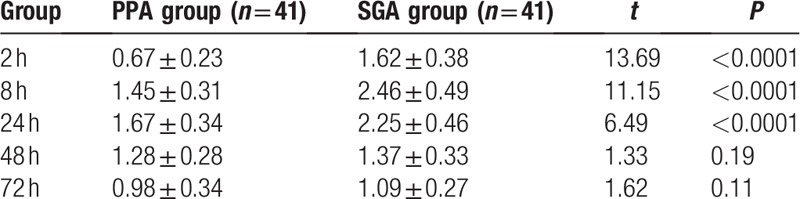
Postoperative VAS scores at different time point in two groups.

### 3.4 Changes of serum VEGF and TGF-β

There was no significant difference in serum levels of VEGF and TGF-β between the two groups before operation. The expression level of VEGF in PPA group was significantly lower than that in SGA group 24 h after operation (*P* < 0.05), and the level of TGF-β in PPA group was also significantly lower than that in SGA group (*P* < 0.05). There was no significant difference in serum levels of VEGF and TGF-β between before and after operation in PPA group. Serum levels of VEGF and TGF-β at 24 h after operation in SGA group were significantly higher than those before operation (*P* < 0.05), as shown in Table 4.

**Table 4:**
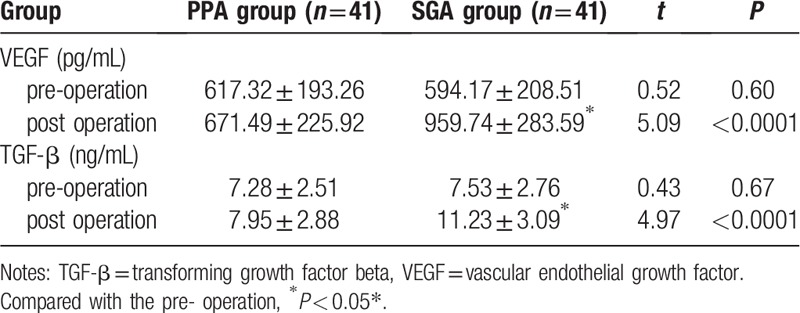
Changes of serum VEGF and TGF-β.

### 3.5 Adverse reactions

There were no complications such as pneumothorax, hematoma and toxic reaction of local anesthetics in PPA group. There was no significant difference in adverse reactions between the two groups.

## 4 Discussions

Surgery is the main method for the treatment of NSCLC. The mode of anesthesia can be divided into general anesthesia or general anesthesia combined with regional anesthesia, but whether the mode of anesthesia is related to the prognosis of malignant tumor patients and which anesthesia is better are still controversial.^[6]^ A systematic review suggested that intravenous general anesthesia is more beneficial to the prognosis of cancer patients than inhaled general anesthesia. Epidural anesthesia was once the golden standard for postoperative thoracic analgesia, but its clinical application was limited because of its rare catastrophic complications.^[7]^ In recent years, with the application of ultrasound equipment in the field of anesthesia, paravertebral nerve block can be performed under the guidance of ultrasound. The analgesic effect is consistent with epidural anesthesia, and the incidence of complications such as hypotension and urinary retention is low. What is more, there is no potential serious intraspinal complications.^[8]^ This study is expected to provide more clinical evidence of thoracic paravertebral nerve block-propofol intravenous general anesthesia in patients undergoing thoracoscopic-assisted radical resection of NSCLC.

Thoracoscopic surgery is less invasive than traditional thoracotomy. We performed paravertebral nerve block by ropivacaine which had a long acting time. Flurbiprofen and analgesic pump were used for analgesia after surgery. In this study, we found that the consumption of analgesics in PPA group was significantly lower than that in SGA group (*P* < 0.05). VAS score of PPA group was significantly lower than that of SGA group at 2, 8, and 24 h after operation (*P* < 0.05), and there were no complications related to paravertebral nerve block. There was no significant difference in VAS scores between the two groups at 48 and 72 h after operation, which was related to the half-life. Thoracic paravertebral nerve block is in line with the concept of enhanced recovery after surgery (ERAS), and is expected to become a new standard for perioperative anesthesia and analgesia in thoracic surgery.^[9]^

Tumorigenesis, development and metastasis depend on angiogenesis. VEGF and TGF-β are the key regulators of angiogenesis and play an important role in the growth and metastasis of tumors.^[10]^ It has been reported that VEGF could promote mitosis of lung cancer endothelial cells to form new blood vessels and increase microvascular permeability.^[11]^ Serum levels of VEGF have relatively high sensitivity and specificity to NSCLC, which can be used as an indicator for diagnosis, prediction of therapeutic response, and efficacy evaluation, and have a significant correlation with progression-free survival and overall survival rate.^[12,13]^ TGF-β can promote the growth of lung cancer cells, which is related to the invasion and metastasis of tumor cells. It may be regarded as a biological marker of lung cancer metastasis.^[14]^ It induces epithelial-mesenchymal transition in NSCLC cells and forms invasive cancer cells.^[15]^ In addition, TGF-β increases the expression of VEGF, which provides favorable conditions for NSCLC metastasis.^[16]^

This study found that there was no significant difference in serum levels of VEGF and TGF-β between the two groups before operation. The expression levels of VEGF and TGF-β in PPA group were significantly lower than those in SGA group 24 h after operation (*P* < 0.05). The results were basically consistent with other studies on the effects of propofol-epidural anesthesia and sevoflurane-opioid anesthesia on serum levels of VEGF and TGF-β in postoperative colon cancer patients.^[17]^ Low levels of VEGF and TGF-β in PPA group may reduce the risk of invasion, proliferation and metastasis of residual lung cancer cells and improve the long-term prognosis of NSCLC. The mechanism of the effect of anesthesia on the concentration of VEGF and TGF-β has not been fully elucidated. Studies have shown that propofol has anti-tumor effects by promoting NK cell toxicity, and sevoflurane promotes the proliferation of human glioma stem cells through hypoxia-inducible factors and promotes the growth of tumors.^[18,19]^ Opioids can promote the growth and metastasis of tumors by regulating cellular and humoral immune responses and activating neuroendocrine-mediated stress response leading to angiogenesis.^[4]^ Local anesthetics inhibit the growth of cancer cells by inhibiting the phosphorylation of intercellular adhesion molecule-1.^[20]^ Pain produces immunosuppressive effects by triggering the sympathetic nervous system and HPA axis and increasing the concentration of β-endorphin. Effective analgesia can reduce the incidence of metastasis in cancer models.^[21]^ Regional block anesthesia can protect NK cell activity by reducing neuroendocrine response mediated by surgery, increase anti-tumor cytokines such as IL-2 and IL-10 and decrease regulatory T cells, TH2 cells, and C-reactive protein, thus protect patients’ immune function to produce anti-tumor effect.^[22]^ Our results may be related to the anesthetics and anesthesia methods.

This study has some limitations. It only included a single participating center, and concentrations of serum VEGF and TGF-β were evaluated before and 24 h after operation. A prospective, randomized, multicenter, and long-range study could provide more definitive evidence to compare the effects of PPA and SGA on the expression of serum VEGF and TGF-β in patients undergoing radical resection of lung cancer.

In conclusion, thoracic paravertebral nerve block-propofol intravenous general anesthesia can reduce the consumption of opioids in patients undergoing radical lung cancer surgery, improve the analgesic effect after operation, and reduce the postoperative serum concentration of VEGF and TGF-β. It is suggested that thoracic paravertebral nerve block-propofol intravenous general anesthesia is more suitable than sevoflurane inhalation general anesthesia for patients undergoing radical resection of NSCLC, which provides a basis for its clinical application.

## Author contributions

**Conceptualization:** Sen Yang.

**Data curation:** Sen Yang, Yu Han.

**Formal analysis:** Xiyang Hu, Yu Han.

**Funding acquisition:** Sen Yang.

**Investigation:** Sen Yang.

**Methodology:** Sen Yang, Xiyang Hu.

**Software:** Xiyang Hu, Yu Han.

**Validation:** Yu Han.

**Writing - Original Draft:** Sen Yang.

**Writing - Review & Editing:** Sen Yang, Xiyang Hu.
